# Long-Term Evolution of Chronic Neuropathic Ocular Pain and Dry Eye Following Corneal Refractive Surgery

**DOI:** 10.3390/jcm14134406

**Published:** 2025-06-20

**Authors:** Cristina Valencia-Sandonís, Amanda Vázquez, Laura Valencia-Nieto, Elena Martínez-Plaza, Marta Blanco-Vázquez, Eva M. Sobas, Margarita Calonge, Enrique Ortega, Amalia Enríquez-de-Salamanca, María J. González-García

**Affiliations:** 1 Institute of Applied Ophthalmobiology (IOBA), Universidad de Valladolid (UVa), Campus Miguel Delibes, Paseo de Belén 17, 47011 Valladolid, Spain; cristina.valencia@uva.es (C.V.-S.); avazquezhs@gmail.com (A.V.); laura.valencia@uva.es (L.V.-N.); elena.martinez.plaza@uva.es (E.M.-P.); marta.blanco.vazquez@uva.es (M.B.-V.); evamaria.sobas@uva.es (E.M.S.); mcalonge@uva.es (M.C.); eortegala@saludcastillayleon.es (E.O.); amalia.enriquez-salamanca@uva.es (A.E.-d.-S.); 2 Pain Unit, Alliance of University Hospitals, Health System of Castilla y León, Calle Dulzaina 2, 47012 Valladolid, Spain; 3 Biomedical Research Networking Center in Bioengineering, Biomaterials and Nanomedicine (CIBER-BBN), Campus Miguel Delibes, Paseo de Belén 17, 47011 Valladolid, Spain

**Keywords:** neuropathic ocular pain, dry eye, refractive surgery, tear biomarkers

## Abstract

**Background/Objectives**: Chronic neuropathic ocular pain (NOP) can manifest concurrently with dry eye (DE) symptoms following ocular surgical procedures. Due to its low prevalence, NOP remains an underrecognized and underdiagnosed postoperative complication, leading to suboptimal management. This study evaluated the long-term evolution of symptoms, signs, and tear biomarkers in patients with NOP and DE after corneal refractive surgery (RS). **Methods**: Patients with chronic NOP and persistent DE-related symptoms after corneal RS were assessed in two visits (V1 and V2), at least two years apart. Symptoms (DE, pain, anxiety, and depression) were measured with specific questionnaires. Clinical examination included a slit-lamp ocular surface evaluation, corneal sensitivity measurement, and subbasal corneal nerve plexus evaluation. Basal tear samples were collected, and a 20-plex cytokine panel and Substance P (SP) were assayed. **Results**: Twenty-three patients (35.57 ± 8.43 years) were included, with a mean time between visits of 4.83 ± 1.10 years. DE symptoms, measured with the Ocular Surface Disease Index questionnaire, improved at V2 (*p* < 0.001), along with a reduction in anxiety and depression levels, measured with the Hospital Anxiety and Depression Scale (*p* = 0.027). Corneal staining also decreased (*p* < 0.001), while subbasal nerve plexus parameters and corneal sensitivity remained unchanged. Tear analysis revealed increased concentrations of fractalkine/CX3CL1 (*p* = 0.039), interleukin (IL)-1 receptor antagonist (Ra) (*p* = 0.025), IL-10 (*p* = 0.002), and SP (*p* < 0.001). **Conclusions**: Symptom improvement may result from better control of underlying pathologies or natural disease progression. However, the increased levels of SP and fractalkine/CX3CL1 suggest sustained neurogenic inflammation, while elevated IL-1Ra and IL-10 indicate a potential compensatory anti-inflammatory response.

## 1. Introduction

Corneal refractive surgery (RS) is one of the most common surgical procedures for the correction of ocular refractive errors. Several techniques are available, but the most commonly performed is the laser-assisted in situ keratomileusis (LASIK) [1]. Although considered a safe procedure with high patient satisfaction rates, the disruption of corneal nerves produced during flap creation in LASIK can lead to complications. Dry eye (DE)-related symptoms are the most frequently reported postoperative complications, with prevalence rates ranging from 36% to 75%. While these symptoms typically improve within 6 to 12 months, up to 50% of patients remain symptomatic at 6 months post-LASIK, with prevalence decreasing to 0.8% after 12 months [2,3].

DE symptoms may be accompanied by ocular pain, further exacerbating the condition. In terms of duration, pain is considered acute if it lasts less than three months, or chronic if it persists for three months or more [4]. From a pathophysiological perspective, this pain may arise from noxious stimulation or ocular surface damage (nociceptive pain) or from the dysfunction of the ocular neurosensory system (neuropathic pain) [5,6,7]. Neuropathic pain is often characterized by pain in response to normally non-painful stimulus (allodynia) and heightened pain from a stimulus that normally provokes less pain (hyperalgesia) [7]. Moreover, neuropathic pain can be further classified as peripheral or central, depending on whether the dysfunction occurs at the level of the ocular surface nerves or involves central processing mechanisms, although this classification is considered controversial [8].

Bertz et al. reported that 11% of patients experience chronic ocular pain after RS [9]. Additionally, Moshifar et al. documented the occurrence of neuropathic ocular pain (NOP) in 1 out of 900 LASIK patients [10]. Our research group has previously reported that chronic NOP afflicted 63.5% of patients seeking consultation with us for persistent symptomatic DE after RS [11]. These conditions substantially reduce patients’ quality of life, affecting daily activities such as reading and driving. Moreover, chronic ocular pain and DE have been linked to sleep disturbances, mental health disorders (e.g., anxiety and depression), and, in severe cases, suicidal ideation [12,13]. Consequently, these complications contribute to decreased work productivity and an increased economic burden, a concern expected to rise with an aging population and the growing number of RS procedures performed worldwide [1,14].

The overlap of pain and DE-related symptoms, combined with the scarcity of clinical signs during ocular surface examination, complicates the identification of pain as a distinct disease, leading to delays in diagnosing and correctly managing NOP [9,10,15]. Standardized diagnostic criteria for NOP have yet to be established. Thus, the clinical evaluation of these patients must be very thorough, beginning with a comprehensive clinical history that considers possible risk factors, such as anxiety and depression, or the presence of DE and/or pain symptoms before RS [9]. The use of standardized and validated questionnaires for symptom assessment is crucial, as these patients often present symptoms disproportionate to ocular surface signs. It is well known that after RS, there is a decrease in corneal nerve density and length, with recovery starting 2 weeks post-procedure [16]. Although corneal nerve density increases, studies conducted 2 to 5 years after surgery indicate that it does not reach preoperative levels [17,18,19]. Similarly, corneal sensitivity decreases after RS [20], and patients with ocular pain and DE have been found to exhibit altered corneal sensitivity, which may be either hypersensitivity or hyposensitivity [21,22]. In addition, our research group has recently reported a reduced nerve density and increased microneuroma density as key indicators for confirming NOP in patients with DE symptoms post-LASIK [22]. Previous studies have also described changes in inflammatory and pain-related molecules in the tears of patients with DE and chronic pain and after RS. Cytokines play a key role in the development and maintenance of neuropathic pain by mediating the activation of immune and glial cells after nerve injury. While their involvement in nociceptive sensitization is well-documented, the exact role of each cytokine remains unclear. Nevertheless, these molecules could serve as promising therapeutic targets for new therapeutic strategies [23].

A proper diagnosis and classification of ocular pain type is essential for guiding appropriate management. In cases of peripheral NOP and severe cases of DE disease, topical treatments with neuromodulatory properties, such as blood derivatives, can support nerve regeneration and help to modulate abnormal neural activity, thereby alleviating symptoms. However, in cases of central NOP, these approaches are often insufficient, and management must target central nervous system modulation through systemic treatments such as tricyclic antidepressants or calcium channel α 2-δ ligands, among many others [8]. Given the lack of highly effective treatments, identifying reliable biomarkers of pain remains crucial to better understanding the underlying mechanisms and to guiding the development of more targeted and effective therapeutic strategies.

Therefore, this study aimed to evaluate the long-term evolution of symptoms, clinical signs, and the changes in inflammation- and pain-related molecules in tears of patients with chronic NOP and DE after RS.

## 2. Materials and Methods

This was a prospective, observational, and single-center study approved by the East Valladolid Health Area Ethics Committee (Valladolid, Spain), and it followed the tenets of the Declaration of Helsinki. Prior to enrolment, all participants were informed of the aims of the study and provided written informed consent.

### 2.1. Patient Sample Description and Study Design

Patients with chronic NOP and persistent DE-related symptoms after having undergone corneal RS were recruited and evaluated during an initial visit (V1) by an ophthalmologist and anesthesiologist specialized in chronic pain; this sample forms part of a previously published study, in which patients were recruited online through a patient association, and all had undergone RS at different centers in Spain [11]. At a follow-up visit (V2), conducted at least two years after V1, their long-term outcomes were evaluated following the same assessment protocol and re-evaluated again by the ophthalmologist. Since many patients lived far from our center, they were followed in their respective cities and medical centers rather than at our institution during the period between visits. Therefore, their management during these years was not standardized. Inclusion criteria at V2 were that patients had taken part in the previously published study; inclusion criteria at V1 required the presence of persistent DE-related symptoms or established DE disease [24] in addition to chronic NOP in at least one eye secondary to corneal RS. Exclusion criteria for V1 and V2 included the following: (1) any ocular surface disease other than DE or chronic ocular pain within the last 3 months; (2) any ocular, periocular, or orbital surgeries (excluding RS) undergone previously or between V1 and V2; (3) a diagnosis of systemic condition with possible ocular involvement within the preceding 3 months; (4) beginning of systemic therapies known to influence ocular surface health during the previous 3 months; (5) lacrimal punctum occlusion initiated in the 3 previous months; (6) use of contact lenses during the 7 days prior to the study visit (if they were contact lens users); or (7) application of any topical ophthalmic treatments or lubricants in the 24 and 12 h, respectively, before the study visit.

Ocular pain was defined as a Numerical Rating Scale (NRS) score and Wong–Baker Faces Pain Rating Scale (WFPRS) score of ≥2 [25,26], and considered chronic if it persisted for ≥3 months [4]. Chronic pain was considered as NOP when patients met at least 3 out of the following 5 diagnostic criteria: (1) evidence of damage or injury to the somatosensory nervous system [27]; (2) minimal signs of corneal staining (Oxford score ≤1) [28]; (3) a report of at least two characteristic descriptors associated with neuropathic pain such as tingling, pins-and-needles sensation, stabbing, shooting, or electric shock-like pain [29,30,31]; (4) altered corneal sensitivity, including allodynia, hyperalgesia, and/or radiating pain [32]; and (5) lack of symptoms relief after topical anesthesia, defined by an anesthetic challenge test result ranging from −2 to +5 [29]. Final confirmation of the NOP diagnosis was performed by an anesthesiologist with expertise in oculofacial pain.

In addition, the presence of DE-related symptoms was established based on an Ocular Surface Disease Index (OSDI) score ≥13, while the diagnosis of stablished DE disease was made when both symptoms and at least two of the following clinical signs were present: (1) tear break-up time (TBUT) ≤7 s; (2) corneal staining score ≥1 (Oxford scale); (3) Schirmer test with anesthesia ≤5 mm in 5 min. The presence of DE-related symptoms and signs was corroborated by an ophthalmologist specialized in ocular surface disease.

All participants underwent evaluation after spending 20 min in our Controlled Environment Laboratory (CELab) (www.visionrd.com/celab/) (accessed on 18 March 2025), where normal environmental conditions were maintained (23 °C, 50% relative humidity, and no localized air flow) [33]. Both visits (V1 and V2) were conducted in accordance with the evaluation protocol described below. A thorough medical history was conducted prior to the clinical evaluation, where information regarding current ocular and systemic treatments for DE and NOP management was collected at V1 and V2. In addition, the presence of defining features of NOP such as allodynia, hyperalgesia, and radiating pain were specifically studied.

### 2.2. Symptoms

The assessment of ocular surface symptoms was conducted using the self-administered OSDI questionnaire and the Modified Single-Item Dry Eye Questionnaire (mSIDEQ). The OSDI classifies the severity of the DE-related symptoms into four categories: asymptomatic (score 0–12), mild (score 13–22), moderate (score 23–32), and severe (score 33–100) [34,35]. The mSIDEQ assesses the frequency of symptoms, including dryness, foreign body sensation, burning, pain, itching, photophobia, and blurred vision (rated on a scale of 0–4), resulting in a total score range of 0 to 28 [36]. To measure the intensity of the patient’s main and most bothersome symptom (if not pain) and pain itself, the NRS and the WFPRS were utilized [25,37,38]. The NRS quantifies from 0 to 10 (0–1: no pain; 2–4 mild; 5–7 moderate; 8–10 severe), while the WFPRS employs six horizontally arranged facial expressions to represent increasing levels of pain (0: no pain; 2: discomfort; 4: mild pain; 6: moderate pain; 8: intense pain; 10: unbearable pain) [39]. Anxiety and depression levels were measured using the Hospital Anxiety and Depression Scale (HADS), a 14-item scale with a total score range of 0–42 [40]. This score is derived from two subscales (anxiety and depression), each ranging from 0 to 21 (0–7: normal; 8–10: borderline; 11–21: existence of a clinical problem) [41,42]. Changes in ocular pain and DE symptoms between both visits were evaluated using the Change in Dry Eye Symptoms Questionnaire (CDES-Q), which consist of two parts: the first (CDES-Q1) assesses whether the patient feels better, worse, or the same, while the second (CDES-Q2) evaluates the degree of improvement or decline on a scale of 0 to 10 [43].

### 2.3. Clinical Examination

A slit lamp examination (SL-D7, Topcon Corporation, Tokyo, Japan) was conducted on both eyes to evaluate the ocular surface.

The severity of bulbar conjunctival hyperemia and blepharitis was assessed according to the Efron scale (range, 0–4) [44].

Tear film stability was evaluated through the fluorescein TBUT test, calculating the average of three consecutive measurements following the instillation of previously moistened sodium fluorescein strips (I-DEW flo, Entod Research Cell UK Ltd., London, UK) [24].

Ocular surface integrity was evaluated 2 min after TBUT through corneal fluorescein staining, using the Brien Holden Vision (BHVI) scale (range, 0–4 for each of the five corneal areas). The total BHVI score was derived from the mean of the scores across the five areas [45,46]. Subsequently, conjunctival staining was measured using lissamine green strips (I-DEW green, Entod Research Cell UK Ltd., London, UK) according to the Oxford scale (range, 0–5) [47].

Corneal sensitivity was measured using both non-contact and contact esthesiometry. A prototype of Belmonte’s gas esthesiometer was used to assess corneal sensitivity thresholds for mechanical and thermal stimuli (heat and cold) following established protocols from our research group [48]. Mechanical sensitivity was also quantified in the central cornea using a Cochet–Bonnet esthesiometer (Luneau Ophthalmology, Chartres, Paris, France), with measurements taken before and after the application of topical anesthetic (1 drop of 0.1% tetracaine and 0.4% oxibuprocaine) (Anestésico Doble Colirio; Alcon Cusí, El Masnou, Spain) following standard protocols (range, 60–0 mm). The longest filament length detected was recorded as the corneal threshold [49,50].

Immediately after Cochet–Bonnet esthesiometry post-anesthesia, the anesthetic challenge test was conducted. The change in the intensity of current ocular symptoms was assessed using the Global Rating of Change (GRC) scale, which measures symptom improvement or worsening on a scale from −5 (completely recovered) to 0 (unchanged) to +5 (much worse) [50]. Patients were categorized based on their GRC scores as follows: scores from −5 to −3 were classified as indicative of predominantly peripheral pain, scores from −2 to −1 as mixed pain, and scores from 0 to +5 as suggestive of predominantly central pain [11].

The Schirmer test, performed under topical anesthesia, was used to assess basal tear production [49].

In vivo confocal microscopy (IVCM) was performed on both eyes using the Heidelberg Retina Tomograph III and the Rostock Cornea Module (Heidelberg Engineering GmbH, Heidelberg, Germany) after the application of topical anesthesia. A sterile disposable TomoCap (Heidelberg Engineering GmbH), with a drop of Viscotears gel (Carbomer 980, 0.2%; Novartis Farmacéutica S.A., Barcelona, Spain) applied to both inner and outer surfaces, was positioned over the microscope objective lens. Participants were instructed to focus on a fixation point directly in front of them, while the tip of the TomoCap was placed in contact with the central cornea to capture the images. Each image had a resolution of 384 × 384 pixels, representing a coronal section of 400 × 400 µm (0.16 mm^2^). One eye was randomly selected, and three good quality, non-overlapping images of the central cornea were analyzed by a masked evaluator. The images were analyzed using the Image J software version 1.54g4 and its NeuronJ plugin (https://imagescience.org/meijering/software/neuronj/) (accessed on 9 October 2024). The following parameters were analyzed: (1) total nerve number (n/mm^2^): sum of nerves per image; (2) nerve density (mm/mm^2^): total nerve length per frame; (3) nerve length (mm/mm^2^): mean nerve length per image; (4) nerve tortuosity: assessed using the Oliveira-Soto and Efron scale (range, 0–4); (5) density of nerve branch points (n/mm^2^): number of nerve bifurcations per image; (6) immune cell density (n/mm^2^): number of immune cells identified as bright hyperreflective bodies with dendritic structures; (7) microneuroma density (n/mm^2^): number of microneuromas identified as irregularly shaped terminal enlargements of subbasal nerve endings; and (8) image reflectivity: index of mean plexus reflectivity or optic densitometry using ImageJ’s histogram function. The mean value of the three images was calculated for each parameter.

### 2.4. Tear Sample Collection and Analysis

Basal tear samples were gently collected from the external canthus using glass capillary micropipettes (Drummond Scientific Co., Broomall, PA, USA), while minimizing reflex tearing as outlined in previous protocols [51]. A 1 μL sample was obtained from a randomly selected eye for cytokine analysis and subsequently diluted (1:10) in a cryotube containing 9 μL of ice-cold Milliplex Cytokine Assay Buffer (Merck Millipore, Burlington, MA, USA). For substance P (SP) analysis, a 2 μL basal tear sample was collected from the contralateral eye and diluted (1:25) in the appropriate SP assay buffer (Cayman Chemical, Ann Arbor, MI, USA). All samples were kept at 4 °C throughout the study visit and promptly frozen at −80 °C until assayed.

The concentration of 20 cytokines in tear samples was simultaneously measured using X-MAP technology with two customized immunobead-based assays: SPR 1549 (for samples in V1) and SPR 2141 (for samples in V2) Custom 20-plex Magnetic Human Cytokine Milliplex MAP panels (Millipore, Merck, MA, USA), in a MAGPIX^®^ equipment (Luminex Corporation, Austin, TX, USA), following the manufacturer’s low-volume protocol, which uses 10 μL of sample or standards per assay, as described previously [52]. The molecules analyzed included the following: epidermal growth factor (EGF), fractalkine/CX3CL1, interleukin (IL)-1β, IL-1 receptor antagonist (Ra), IL-2, IL-4, IL-6, IL-8/CXCL8, IL-9, IL-10, IL-17A, monocyte chemoattractant protein (MCP)-1/CCL2, MCP-3/CCL7, tumor necrosis factor (TNF)-α, interferon (IFN)-γ, growth related oncogene (GRO), macrophage inflammatory protein (MIP)-1α/CCL3, MIP-1β/CCL4, nerve growth factor (NGF), and regulated on activation normal T cell expressed and secreted (RANTES)/CCL5. Cytokine concentrations (pg/mL) were calculated based on fluorescence intensity using standard curves, following the protocol previously described [51]. The minimum detectable concentrations (pg/mL) used are presented in Supplementary Table S1.

SP levels in tears were measured using a competitive enzyme-linked immunosorbent assay (ELISA) (Cayman Chemical, Ann Arbor, MI, USA) following the manufacturer’s protocol as previously described [51].

### 2.5. Statistical Analysis

Data were statistically analyzed using SPSS software version 26.0 (SPSS Inc., Chicago, IL, USA). Comparisons were made between both visits. One eye (same for V1 and V2) was randomly selected for the data analysis. Quantitative data were reported as mean ± standard deviation (SD), and qualitative variables were expressed as percentage. For ordinal variables, the median and interquartile range (IQR) were used to summarize distributions. The Shapiro–Wilk test was applied to check the normality assumption.

For comparisons between visits, normally distributed quantitative variables were analyzed using the paired Student’s *t*-test, with Levene’s test used to verify homogeneity of variance. Non-normally distributed, ordinal, or qualitative variables were analyzed using the Wilcoxon signed-rank test.

For molecules with a detection rate of 50% or higher, non-detected values were imputed with the minimum concentration value of the corresponding standard curve. The concentrations of these were log-transformed (log 2) to normalize the distribution and analyzed quantitatively. Those molecules with detection rates below 50% were treated as binary outcomes (detected/non-detected) and analyzed using the McNemar test.

*p*-values ≤ 0.05 were considered statistically significant.

## 3. Results

### 3.1. Sample Description

This is a subset of a group of patients that attended a previous study (V1) that included 66 patients with NOP. All participants were invited to attend the follow-up visit (V2) at the beginning of the second phase of the study. Since they lived in different regions of Spain, often far from our center, they returned according to their personal availability. As a result, the interval between V1 and V2 averaged 4.83 ± 1.10 (range 2.83–7.00) years. A total of 23 patients (14 women and 9 men) attended V2, with a mean age of 35.57 ± 8.43 (range 25–56) years. At V1, all the patients presented DE-related symptoms or established DE disease and chronic NOP secondary to RS. However, at V2 these criteria were not reassessed, as participants from the previous study were invited to participate regardless of their current situation. All patients had undergone RS in both eyes, with a mean of three (range 2–6) procedures in the eye selected for analysis. Most patients, 20 (86.96%), had undergone laser-assisted in situ keratomileusis (LASIK), while 3 (13.04%) had photorefractive keratectomy. Symptoms of ocular pain and DE were reported to have started 10.25 ± 5.20 (range, 3.17–20.17) years before V2. In addition, two patients were rigid gas-permeable contact lens wearers: one used a corneal rigid gas-permeable contact lens and the other a scleral one.

Table 1 summarizes the ocular treatments used to manage DE-related symptoms by patients at V1 and V2. Similarly, Table 2 presents the systemic treatments employed for the management of NOP at both visits. Of the three (13%) patients who reported using other treatments for the management of NOP at V2, these included self-hypnosis, botulinum toxin, and beta-blockers. The treatments listed in Table 1 and Table 2 correspond to those patients were using at the time of V1 and V2. However, there was no follow-up between visits, as the study was not interventional. 

### 3.2. Symptoms

The results from questionnaires assessing DE-related symptoms, anxiety, and depression levels are summarized in Table 3. Patients showed an improvement in DE-related symptoms (evaluated with OSDI), the intensity of their principal main symptom (evaluated with NRS and WFPRS), as well as in anxiety and depression levels.

Regarding the main symptoms, dryness and pain were the most reported ones by patients (Table 3), followed by halos (4.3%) and eye pressure sensation (4.3%) at V1, and by foreign body sensation (13.0%), tightness (4.3%), night glare (4.3%), photophobia (4.3%), itching (4.3%), and burning (4.3%) at V2.

### 3.3. Clinical Examination

Ocular surface slit-lamp evaluation only showed a significant improvement in corneal staining in V2 compared to V1, as shown in Table 4.

The assessment of non-contact corneal sensitivity, performed with Belmonte’s esthesiometer, revealed no significant differences in mechanical and cold thresholds between V1 and V2. Similarly, contact corneal sensitivity, assessed with the Cochet–Bonnet esthesiometer, showed no significant differences between V1 and V2, either before or after topical anesthesia instillation (Table 5).

The analysis of IVCM images revealed no significant differences in parameters between V1 and V2 (Table 6).

### 3.4. Tear Levels of Neuropathic and Inflammatory Pain-Related Molecules

EGF, IL-1Ra, IL-8/CXCL8, MCP-1/CCL2, GRO, and SP were detected in at least 90.5% of the subjects and Fractalkine/CX3CL1, IL-4, and IL-10 were detected in variable ranges from 60% to 100% of the subjects (Table 7). Detection rates for IL-1β, IL-2, IL-6, IL-9, IL-17A, MCP-3/CCL7, TNF-α, IFN-γ, MIP-1α/CCL3, MIP-1β/CCL4, NGF, and RANTES/CCL5 were below 50% in at least one visit and were therefore analyzed qualitatively.

**Figure 1:**
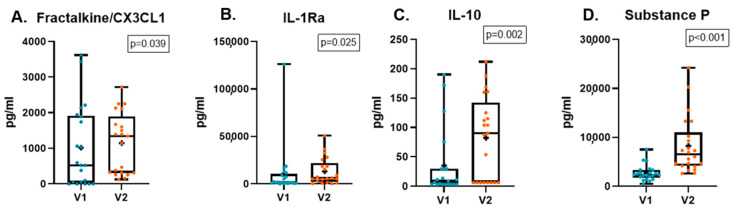
Changes in the concentration of Fractalkine/CX3CL1 (**A**), IL-1Ra (**B**), IL-10 (**C**), and substance P (**D**) between V1 and V2. Data are presented as mean concentrations (pg/mL) along with the 25th and 75th percentiles. Each dot represents an individual value, and the cross indicates the mean value.

Table 8 shows the changes in tear concentration of quantitatively analyzed molecules. Comparing V1 and V2, Fractalkine/CX3CL1, IL-1Ra, IL-10, and SP concentrations significantly increased at V2 (Figure 1).

Regarding the molecules analyzed qualitatively, there was found an increased detection rate (Table 7) of IL-2, IL-9, IL-17A, and MCP-3/CCL7 in V2 compared to V1 (*p* = 0.008, *p* = 0.039, *p* < 0.001 and *p* = 0.006, respectively). In contrast, the detection rate of NGF in V2 was lower than in V1 (*p* < 0.001).

## 4. Discussion

Chronic ocular pain and persistent DE-related symptoms are highly disabling conditions that can appear following RS. These conditions can significantly interfere with daily activities, leading to a reduced quality of life and an increased risk of mental health issues [2,12,21]. The overlap between ocular pain, particularly NOP, and DE-related symptoms complicates the recognition of pain as a distinct condition and as a disease in itself [53]. However, early identification of ocular pain is crucial for optimal management and prevention of central sensitization [11]. To date, most studies have primarily focused on characterizing pain and DE in the immediate and short-term postoperative periods, up to 2–5 years. To our knowledge, this is the first study to analyze the long-term evolution of symptoms, clinical signs and inflammation- and pain-related molecules in tears of patients with NOP and persistent DE-related symptoms following RS.

Of the 66 patients included in the previous study (V1), 23 patients decided to return for V2. The low recruitment rate is due to the fact that they lived far from our center and, therefore, decided not to return for the second visit. Our results showed a significant improvement in the intensity of DE-related symptoms, whereas pain levels remained similar between both visits. Patients in our sample reported moderate pain and severe dryness symptoms at both visits, indicating a symptomatology disproportionate to the clinical signs observed on the ocular surface. These findings are consistent with previous studies, which have demonstrated a positive correlation between pain levels and DE symptom scores, highlighting the substantial overlap and interrelation between these symptoms [9,25,54].

Beyond dryness, additional symptoms such as photophobia, itching, night glare, and pain were reported in our sample. While pain has historically been seen under the scope of DE and therefore treated as such, the importance of accurate phenotyping is now widely recognized to ensure the most appropriate treatment, particularly in cases of NOP [11,12]. NOP represents the most challenging type of ocular pain to manage, requiring highly specific, targeted treatments that address the underlying pain mechanisms [12]. Although pain intensity remained consistently moderate across visits, an improvement in DE-related symptoms, as evaluated with OSDI questionnaire, was observed at V2, along with a reduction in the intensity of the main symptom. This suggests that while DE symptoms improved, the management of pain remained a challenge in this sample of patients. The management of these patients evolved from ocular lubrication at V1 to a combination of lubrication and cyclosporine at V2. However, it is important to note that treatment data was only collected at the time of visits, without accounting for the years between visits, and adherence to treatment was not assessed. The lack of improvement in pain symptoms could be attributed to the nature of NOP, which often does not respond to standard DE therapies [15], and to the absence of targeted pain treatments. Systemic treatments targeting NOP are frequently discontinued due to the lack of rapid symptom relief [13]. Patients also exhibited high levels of anxiety and depression, which were significantly reduced at V2. This aligns with previous findings that suggest persistent NOP and DE symptoms after RS are associated with mental health issues, including anxiety, depression, and, in severe cases, suicidal ideation [10,11,13]. This improvement could be attributed to the fact that by the time of the second visit, patients were diagnosed several years ago. Recognizing pain as a chronic condition enables patients to better understand and manage their situation [55]. Furthermore, addressing their expectations and explaining the complexities of pain remission also play a key role in their coping process, promoting a sense of recognition and validation, and ensuring continuous follow-up of their condition. However, it is important to note that there was no follow-up between visits, and psychological treatments were not considered in this study.

Clinically, this study found a significant reduction in corneal staining, although the degree of staining was not clinically relevant. No significant differences were observed in either the morphology of the corneal subbasal nerve plexus or corneal sensitivity, suggesting that these parameters did not fully recover even after an average of 11 (range 3–7) years after LASIK in patients who developed chronic NOP and DE-related symptoms. Corneal nerve density decreases immediately after LASIK, having the lowest density at five days postoperatively, followed by a gradual recovery [19]. Some studies have reported a return to preoperative levels of corneal innervation within 12 months in patients without postoperative complications [17,54]. However, other studies have shown that corneal innervation does not return to preoperative levels, as evidenced by follow-up periods of 6, 12, and 24 months after surgery [19,56]. Only one study has evaluated corneal innervation 10 years after RS, reporting that nerve length, tortuosity, and reflectivity gradually returned to normal values, while nerve density remained unchanged [57]. Subbasal corneal nerve parameters have been positively correlated with corneal sensitivity, which helps explain the decline in sensitivity observed postoperatively [56]. Corneal sensory innervation damage present in DE and/or ocular pain could also lead to corneal hypoesthesia [58], although another study reported corneal hyperesthesia in patients with DE [59].

Regarding the inflammation- and pain-related molecules evaluated in tears, our results revealed a significant increase in the tear concentration of Fractalkine/CX3CL1, IL-1Ra, IL-10 and SP. Fractalkine/CX3CL1 is a potent attractant for immune cells and plays a crucial role in neuroimmune signaling. Its sustained upregulation has been implicated in pain facilitation by promoting neuroinflammation and central sensitization [60,61]. Given these properties, the elevated fractalkine levels observed in our patients may contribute to the persistence of NOP and DE more than a decade after corneal RS. No significant differences in Fractalkine/CXC3CL1 levels were found in healthy individuals after RS compared to those with chronic ocular pain and DE after RS [51,62]. However, higher concentrations of Fractalkine/CX3CL1 have been observed in patients with DE disease [52]. IL-1Ra is a cytokine that contributes to resolving the inflammatory response. Higher IL-1Ra concentration has been found in DE disease patients. In addition, negative correlation was found with TBUT and Schirmer test [52]. The observed increase in IL-1Ra level in our study suggests the presence of neurogenic inflammation, similar to what has been described in neuropathic pain models. This type of inflammation, mediated by proinflammatory cytokines and chemokines, may contribute to corneal nerve sensitization and the persistence of chronic ocular pain following refractive surgery [61].

On the other hand, IL-10 is a cytokine known for its anti-inflammatory and immunosuppressive effects, regulated by other cytokines such as IL-4, IL-13, and IFN-γ, as well as through autoregulation [63]. The increase in IL-10 tear levels observed in our patients may represent a compensatory response to the persistent inflammation. In the context of the eye, IL-10 has been previously found to be elevated postoperatively in both asymptomatic patients and those with DE and pain [51]. Higher tear IL-10 levels were found in patients with Sjögren and non-Sjögren DE disease [52,64]. Some researchers suggest that IL-10 acts as a key cytokine with analgesic properties [63]. While our findings and previous studies suggest a link between IL-10 and ocular conditions, the precise role of IL-10 in the development and maintenance of NOP and DE remains to be fully elucidated. Further research is necessary to confirm these observations and explore the underlying mechanisms.

SP is a neuropeptide that contributes to neurogenic inflammation and in the modulation of pain pathways [65,66]. Several studies have found higher SP levels in patients after RS with and without DE and pain-related symptoms up to 12 months after the procedure [51,56]. Previous studies have linked reduced corneal nerve density to increased tear SP concentration, suggesting that the release of this neuropeptide may be associated with the process of corneal reinnervation [17,56].

Our results also revealed an increase in V2 in the percentage of detection of IL-2, IL-9, IL-17A and MCP-3/CCL7, while NGF detection rate decreased. However, due to the low detection levels of these molecules, they were analyzed only qualitatively as either detected or non-detected, but not quantitatively. Our research group found higher levels of IL-2 and IL-17A in tears of patients 6 months after advanced surface ablation RS compared to preoperative levels and also found a significant effect of time on IL-2 and IL-17A tear concentration [62]. However, in that study, contrary to our results, they were detected in sufficient quantity to study their concentration; this could be due to the exacerbated anti-inflammatory response that patients still present 6 months after corneal surgery, which may be related to the ongoing healing process. IL-9 is a proinflammatory cytokine, previously associated with RS and ocular pain [51,62,67], and MCP-3/CCL7 is a chemokine related to neuropathic pain that has also been found to be highly expressed in the conjunctiva of patients with vernal keratoconjunctivitis [68,69,70]. NGF is a neurotrophic factor that has been previously associated with corneal nerve regeneration after surgery [71]. The observed changes in the detection rates of these molecules align with prior findings, suggesting a potential involvement of IL-2, IL-9, MCP-3/CCL7, and IL-17A in a sustained inflammatory response following RS and DE, while the decreased NGF detection may reflect that there is no longer evidence of active nerve regeneration in these patients.

This study had some limitations. Firstly, the absence of a control group prevented monitoring of the natural effect of time. Secondly, systemic and ocular treatments followed during the years between visits were not monitored. Patients included in the study came from different parts of Spain, so it was impossible to plan a study with different follow-up visits. Therefore, the effect of pain treatment could not be identified. Thirdly, although evaluations at V1 and V2 were performed by two different observers, they were trained using the same standardized protocol by the same experienced clinician, in order to minimize inter-observer variability. Although IVCM analysis was conducted by a single observer, the previous study using the same population of patients demonstrated excellent inter-observer reliability (intraclass correlation coefficient > 0.90) for all nerve parameters, except for tortuosity, which showed moderate agreements (intraclass correlation coefficient = 0.63) [22]. Moreover, the relatively small number of participants who completed both visits, due to a high rate of loss to follow-up, may limit the strength and generalizability of the findings. Finally, the observational nature of the study does not allow establishing causal relationships. One of the main weaknesses of the study was the inability to correlate the observed changes with specific treatments or with the natural progression of the disease.

Despite these limitations, this study provides valuable insights into the changes that occur in the tear film composition of patients with NOP and DE-related symptoms or DE disease, even in the absence of observable clinical changes. Our findings contribute to a better understanding of the molecular alterations associated with these conditions. This molecular profiling could be instrumental in identifying specific biomarkers for disease monitoring and may support the development of more targeted and effective treatments. The finding that corneal nerve alterations and sensitivity impairments persist for over a decade post-LASIK reinforces the concept that chronic NOP is a long-standing condition.

This study has several potential implications for clinical practice, particularly in the management of patients with chronic NOP and DE-related symptoms post-LASIK. Clinicians should distinguish between nociceptive and neuropathic pain components in DE, incorporating more comprehensive assessments, and implement more comprehensive assessments and the use of neuropathic pain screening tools in routine DE evaluations. While nociceptive pain must be managed by ophthalmologists, neuropathic pain needs a multidisciplinary approach, integrating at least ophthalmologists, anesthesiologists experienced in pain, and psychologists specialized in pain management.

In summary, this study may change clinical practice by recognizing chronic NOP as a distinct, long-term condition and promoting a more nuanced, multidisciplinary approach to managing chronic DE-related symptoms and NOP, emphasizing long-term follow-up, accurate diagnosis, management of neurogenic inflammation, and addressing mental health comorbidities.

## 5. Conclusions

In conclusion, this observational study describes the real-life evolution of patients with chronic NOP and DE-related symptoms over time, under conditions that reflect routine clinical practice. An improvement in symptomatology (symptoms of DE, anxiety, and depression) was observed. This may be attributed to the better control of the underlying pathologies due to the treatment received between the two visits, an accurate diagnosis, or the clinical evolution of the disease. The lack of improvement in pain intensity could be attributed to the neuropathic nature of the pain in these patients. Corneal subbasal nerve plexus and corneal sensitivity did not fully recover, even after an average of 11 years post-LASIK, in patients with chronic NOP and DE-related symptoms. The observed increase in SP and Fractalkine/CX3CL1 tear concentration over time suggests a sustained involvement of neurogenic inflammation in the pathophysiology of NOP. Additionally, the increase in IL-1Ra and IL-10 levels implies a potential compensatory anti-inflammatory/protective response in these patients.

## Figures and Tables

**Table 1:** Ocular topical treatments used at V1 and V2 for the management of dry eye-related symptoms.

	V1 *n* (%)	V2 *n* (%)	*p*-Value
Lubrication during day	23 (100)	18 (78.3)	0.063
Lubrication at bedtime	15 (65.2)	6 (26.1)	**0.004**
Lid hygiene	2 (8.7)	8 (34.8)	**0.031**
Cyclosporine	6 (26.1)	10 (43.5)	0.289
Blood derivatives	5 (21.7)	5 (21.7)	1.000
Corticosteroids	0 (0)	2 (8.7)	0.500

Data are presented as frequencies *n* (%). V1: visit 1; V2: visit 2. *p*-values in bold indicate a statistically significant change from V1 to V2.

**Table 2:** Systemic treatments used at V1 and V2 for the management of neuropathic ocular pain.

	V1 *n* (%)	V2 *n* (%)	*p*-Value
Tricyclic antidepressants	1 (4.3)	3 (13.0)	0.500
Inhibitors of neuronal serotonin and norepinephrine reuptake	1 (4.3)	3 (13.0)	0.625
Calcium channel α 2-δ ligands	0 (0)	2 (8.7)	0.500
Anticonvulsants	1 (4.3)	1 (4.3)	1.00
Analgesics	4 (17.4)	2 (8.7)	0.625
Anxiolytics and antidepressants	7 (30.4)	4 (17.4)	0.375
Other	0 (0)	3 (13.0)	0.250

Data are presented as frequencies *n* (%). V1: visit 1; V2: visit 2.

**Table 3:** Changes in symptomatology.

	V1	V2	*p*-Value
CDES-Q1			
Better *n* (%)	-	16 (69.6)	-
Worse *n* (%)	-	5 (21.7)	-
Same *n* (%)	-	2 (8.7)	-
CDES-Q2			
Improvement intensity (0–10)	-	6.13 ± 2.33	-
Worsening intensity (0–10)	-	5.40 ± 3.21	-
OSDI (0–100)	62.12 ± 18.70	48.52 ± 24.24	**<0.001**
mSIDEQ (0–28)	19.96 ± 4.28	17.43 ± 5.78	0.066
Pain as main symptom *n* (%)	8 (34.7%)	4 (17.3%)	0.125
Dryness as main symptom *n* (%)	13 (56.5%)	10 (43.5%)	0.453
Main symptom intensity (NRS, 0–10)	7.48 ± 2.13	6.04 ± 2.17	**0.026**
Main symptom intensity (WFPRS, 0–10)	7.39 ± 2.29	5.96 ± 2.18	**0.036**
Intensity of pain (NRS, 0–10)	6.39 ± 2.10	6.17 ± 3.12	0.759
Intensity of pain (WFPRS, 0–10)	6.17 ± 2.17	5.78 ± 2.92	0.558
HADS questionnaire (0–42)	20.48 ± 7.87	16.13 ± 9.70	**0.027**
Anxiety subscale (0–21)	11.39 ± 3.76	9.26 ± 5.38	**0.039**
Depression subscale (0–21)	9.09 ± 4.63	6.87 ± 4.70	**0.033**

Data are presented as mean ± standard deviation or as frequencies. V1: visit 1; V2: visit 2; CDES-Q: Change in Dry Eye Symptoms Questionnaire; OSDI: Ocular Surface Disease Index; mSIDEQ: Modified Single Item Dry Eye Questionnaire; NRS: Numerical Rating Scale; WFPRS: Wong–Baker Faces Pain Rating Scale; HADS: Hospital Anxiety and Depression Scale. *p*-values in bold indicate a statistically significant change from V1 to V2.

**Table 4:** Changes in clinical test results.

	V1	V2	*p*-Value
Conjunctival hyperemia (Efron scale, 0–4)	2.00 [2.00–1.00]	2.00 [2.00–1.00]	0.919
Blepharitis (Efron scale, 0–4)	1.00 [1.00–0.00]	1.00 [1.00–0.00]	0.905
TBUT (seconds)	4.51 ± 2.92	3.85 ± 2.13	0.626
Corneal staining (BHVI, 0–4)	0.91 ± 0.77	0.45 ± 0.63	**<0.001**
Conjunctival staining (Oxford scale; 0–5)	0.65 ± 0.88	0.30 ± 0.56	0.072
Schirmer test (mm)	8.91 ± 10.49	9.04 ± 10.02	0.882

Data are presented as mean ± standard deviation. V1: visit 1; V2: visit 2; TBUT: tear break-up time; BHVI: Brien Holden Vision Institute scale. *p*-values in bold indicate statistically significant changes from V1 to V2.

**Table 5:** Corneal sensitivity changes.

	V1	V2	*p*-Value
Non-contact corneal esthesiometry			
Mechanical threshold (ml/min)	120.00 ± 42.52	119.47 ± 53.41	0.973
Heat threshold (°C)	0.86 ± 0.89	1.02 ± 1.08	0.654
Cold threshold (°C)	−1.58 ± 1.38	−1.40 ± 1.19	0.645
Contact corneal esthesiometry			
Before topical anesthesia (mm)	55.87 ± 7.49	56.96 ± 7.65	0.413
After topical anesthesia (mm)	8.91 ± 16.99	15.43 ± 18.09	0.110
Anesthetic challenge test (GRC scale; −5 to 5)	−0.70 ± 2.48	−1.63 ± 2.14	0.053
Peripheral pain (GRC scale; −5 to −3)	7 (30.4)	11 (47.8)	0.289
Mixed pain (GRC scale; −2 to −1)	4 (17.4)	3 (13.0)	1.000
Central pain (GRC scale; 0 to 5)	12 (52.2)	9 (39.1)	0.453

Data are presented as mean ± standard deviation or frequencies. V1: visit 1; V2: visit 2; GRC: Global Rating Scale.

**Table 6:** Changes in subbasal corneal nerve plexus parameters.

	V1	V2	*p*-Value
Number of nerves (n/mm^2^)	29.27 ± 16.13	37.22 ± 24.99	0.186
Nerve density (mm/mm^2^)	7103.00 ± 3883.26	9625.21 ± 6101.39	0.156
Nerve length (mm/mm^2^)	1580.35 ± 304.28	1641.02 ± 408.60	0.570
Density of nerve branch points (n/mm^2^)	12.56 ± 13.91	16.39 ± 22.64	0.370
Nerve tortuosity (0–4)	2.40 ± 1.14	2.48 ± 0.97	0.848
Density of microneuromas (n/mm^2^)	0.97 ± 1.83	0.69 ± 1.70	0.709
Density of dendritic cells (n/mm^2^)	62.06 ± 101.37	61.94 ± 81.88	0.996
Reflectivity	100.75 ± 12.36	104.13 ± 11.97	0.467

Data are presented as mean ± standard deviation. V1: visit 1; V2: visit 2.

**Table 7:** Percentage of detection of the 20 cytokines and substance P analyzed in V1 and V2.

	V1	V2
EGF	100	100
Fractalkine/CX3CL1	76.2	100
IL-1β	*28.6*	*33.3*
IL-1Ra	95.2	100
IL-2	*14.3*	52.4
IL-4	76.2	85.7
IL-6	*38.1*	57.1
IL-8/CXCL8	95.2	90.5
IL-9	*22.2*	61.1
IL-10	60.0	65.0
IL-17A	*0*	57.1
MCP-1/CCL2	95.2	100
MCP-3/CCL7	*47.6*	95.2
TNF-α	*14.3*	*23.8*
IFN-γ	*33.3*	52.4
GRO	100	100
MIP-1α/CCL3	*23.8*	*33.3*
MIP-1β/CCL4	*33.3*	57.1
NGF	90.5	*28.6*
RANTES/CCL5	*42.9*	57.1
Substance P	100	100

Data are presented as percentages. Molecules with a percentage of detection lower than 50% are in italics. V1: visit 1; V2: visit 2; EGF: epidermal growth factor; IL: interleukin; IL-1Ra: interleukin-1 receptor antagonist; MCP: monocyte chemoattractant protein; TNF: tumor necrosis factor; IFN: interferon; GRO: growth related oncogene; MIP: macrophage inflammatory protein; NGF: nerve growth factor; RANTES: regulated on activation normal T cell expressed and secreted.

**Table 8:** Concentrations (pg/mL) of tear molecules analyzed in V1 and V2.

Molecule	V1	V2	*p*-Value
EGF	2315.14 (1695.81–2934.47)	1632.14 (1163.19–2101.10)	0.302
Fractalkine/CX3CL1	1014.28 (491.29–1537.27)	1146.33 (761.35–1531.32)	**0.039**
IL-1Ra	10,580.91 (−1739.03–22,900.86)	13,135.42 (6688.63–19,582.23)	**0.025**
IL-4	344.26 (161.12–527.40)	370.33 (221.46–519.20)	0.624
IL-8/CXCL8	171.94 (60.66–283.21)	226.86 (73.23–380.49)	0.971
IL-10	35.04 (9.03–61.06)	82.40 (50.24–114.56)	**0.002**
MCP-1/CCL2	371.98 (202.22–541.74)	289.58 (147.66–431.50)	0.770
GRO	3319.00 (2209.59–4428.41)	3087.00 (2044.84–4129.16)	0.940
Substance P	2712.34 (2005.06–3419.62)	8232.89 (5657.59–10,808.18)	**<0.001**

Data is shown as mean concentration (95% confidence interval) pg/mL. V1: visit1; V2: visit 2; NA: not applicable; EGF: epidermal growth factor; IL: interleukin; IL-1Ra: interleukin-1 receptor antagonist; MCP: monocyte chemoattractant protein; GRO: growth related oncogene; NGF: nerve growth factor; RANTES: regulated on activation normal T cell expressed and secreted. *p*-values in bold indicate statistically significant changes from V1 to V2.

## Data Availability

The original contributions presented in this study are included in the article. Further inquiries can be directed to the corresponding author.

## Supplementary Materials

The following supporting information can be downloaded at https://www.mdpi.com/article/10.3390/jcm14134406/s1, Table S1: Minimum detectable concentrations (pg/mL) of standard curves.

## Abbreviations

The following abbreviations are used in this manuscript:

RS	Refractive surgery
LASIK	Laser-assisted in situ keratomileusis
DE	Dry eye
NOP	Neuropathic ocular pain
V1	Visit 1
V2	Visit 2
NRS	Numerical Rating Scale
WFPRS	Wong-Baker Faces Pain Rating Scale
CELab	Controlled Environment Laboratory
OSDI	Ocular Surface Disease Index
mSIDEQ	Modified Single-Item Dry Eye Questionnaire
HADS	Hospital Anxiety and Depression Scale
CDES-Q	Change in Dry Eye Symptoms Questionnaire
TBUT	Tear Break-up Time
BHVI	Brien Holden Vision Institute
GRC	Global Rating of Change
IVCM	In vivo confocal microscopy
SP	Substance P
EGF	Epidermal growth factor
IL	Interleukin
IL-1Ra	Interleukin-1 receptor antagonist
MCP	Monocyte chemoattractant protein
TNF	Tumor necrosis factor
IFN	Interferon
GRO	Growth related oncogene
MIP	Macrophage inflammatory protein
NGF	Nerve growth factor
RANTES	Regulated on Activation Normal T cell Expressed and Secreted
ELISA	Enzyme-linked immunosorbent assay
SD	Standard deviation
IQR	Interquartile range
