# Supplementary material for: Long-Term Evolution of Chronic Neuropathic Ocular Pain and Dry Eye Following Corneal Refractive Surgery
Source: J Clin Med. 2025 Jun 20;14(13):4406. doi: 10.3390/jcm14134406 (PMC12249925; doi:10.3390/jcm14134406)
Supplement: Supplementary file 1 [file jcm-14-04406-s001.zip › jcm-3608501-supplementary.docx]

Supplementary Table S1. Minimum detectable concentrations (pg/ml) of standard curves.

|  | V1 | V2 |
| --- | --- | --- |
| EGF | 0.32 | 0.61 |
| Fractalkine/CX3CL1 | 0.32 | 0.61 |
| IL-1β | 0.80 | 0.61 |
| IL-1Ra | 0.32 | 0.61 |
| IL-2 | 0.32 | 0.61 |
| IL-4 | 0.32 | 0.61 |
| IL-6 | 0.80 | 1.53 |
| IL-8/CXCL8 | 0.64 | 1.23 |
| IL-9 | 0.32 | 0.61 |
| IL-10 | 0.32 | 0.61 |
| IL-17A | 0.32 | 0.61 |
| MCP-1/CCL2 | 0.96 | 1.85 |
| MCP-3/CCL7 | 0.32 | 0.61 |
| TNF-α | 0.32 | 1.23 |
| IFN-γ | 0.32 | 0.61 |
| GRO | 0.32 | 0.61 |
| MIP-1α/CCL3 | 0.32 | 0.61 |
| MIP-1β/CCL4 | 0.32 | 0.61 |
| NGF | 0.32 | 0.61 |
| RANTES/CCL5 | 0.32 | 0.61 |

V1: visit 1; V2: visit 2; EGF: epidermal growth factor; IL: interleukin; IL-1Ra: interleukin-1 receptor antagonist; MCP: monocyte chemoattractant protein; TNF: tumor necrosis factor; IFN: interferon; GRO: growth related oncogene; MIP: macrophage inflammatory protein; NGF: nerve growth factor; RANTES: regulated on activation normal T cell expressed and secreted.
